# A Text-Book of Practical Medicine

**Published:** 1901-05

**Authors:** 


A Text-Book of Practical Medicine.—By William Gilman Thompson, M. D., Professor of Medicine in Cornell University Medical College, New York City, Physician to the Presbyterian and Bellevue Hospitals, New York. In one magnificent octavo volume of 1010 pages, with 79 engravings. Cloth, $5.00, net; leather, $6.00, net; half Morocco, $6.50, net. Lea Brothers & Co., Publishers. 1900.

Dr. Thompson's position as a teacher and practitioner should give the profession a work of great value. He has covered the entire field of modern medicine, and has proceeded upon the correct assumption that the proper object of medical science is to cure or aid the sick and prevent disease. This book embraces all of the advantages of definite knowledge, such as has recently come to us through modern pathology, clinical microscopy and bacteriology. Curative medicine has been made a prominent feature throughout the volume. The practitioner will appreciate this work as being not only scientific, but eminently practical. As a single volume book it has no superiors. T. J. B.

